# Re: Long term outcomes of one-stage augmentation anterior urethroplasty: a systematic review and meta-analysis

**DOI:** 10.1590/S1677-5538.IBJU.2021.0856

**Published:** 2022-01-10

**Authors:** Wojciech Perdzynski

**Affiliations:** 1 Damian's Hospital Warsaw Poland Damian's Hospital - Reconstructive Surgery of the Male Genito-Urinary Tract, Warsaw, Poland

*To the editor,*

Authors reviewed the current literature on augmentation anterior urethroplasty choosing for a detailed analysis of large groups of patients from leading centres in which long-term postoperative observation time is performed. They hypothesized that good long-term results are far below the commonly quoted 85%. In conclusion, the authors stated that the success of augmentation urethroplasty in the long-term (after more than 100 months of follow-up) appears to keep deteriorating (1). Very exact statistics supported authors' opinion that augmentation urethroplasty has worst than expected long term results (1). This important publication reminded us from the beginning of 21 century (2-4) when there were descriptions of stricture recurrence rate after buccal mucosa graft urethroplasty (BMGU) done for urethral stricture (US) that increased every 5 years from the operation, reaching 30% or even more 15 years after the operation. Those publications stimulated us to think about finding the cause of deterioration in so many patients with a long-term follow-up and to think about a treatment for that complication. In all patients who were sent to our Department with re-stricture after operations for US done elsewhere, hard scars along penile urethra could be palpated. We came to the conclusion that a treatment to decrease scar formation may be a solution for US recurrence. It seemed to us reasonable to try to prevent those complications actively instead of using “watch and see” passive method of postoperative observation and waiting whether recurrence of US appears or not. That is why in 2010 in a prospective study we introduced a calibration (not dilatation) of urethra with anti-scar gel as a preventive method for stricture recurrence (5). We began to act actively during the time of scar remodelling which starts early in postoperative time and is followed by scar contraction, the main cause -, as we hypothesized, - of US recurrence. We used only calibrations: we introduced dilator the same size as the actual inner size of urethra. That excluded the possibility of urethral injury, which may occur during dilatations. All patients described in our paper had had many urethral instrumentations such as dilatations and endoscopic incisions and reoperations for US. We decided to perform BMGU and 5 weeks later, after internal urethral caliber was measured (by calibrator) we started calibrations with anti-scar gel performed by patients themselves (after teaching them how to disinfect calibrators and to perform self-calibration) under our supervision (usually every six weeks in the first year after operation). We are convinced that the best results of that treatment can be obtained during early remodelling phase of postoperative wound healing in which anti-scar gel can prevent fibrosis and in consequence prevent scar contracture. In our publication, phases of wound healing are described as well as action of anti-scar gel, which may help to understand a scientific background of our method of treatment. Until now, in 39 patients with recurrent anterior US (31 of them had failure after hypospadias repair) calibration with anti-scar gel was used with good results. Median follow-up (at the time of publication) was 61 months (range 12 to 114). In uroflowmetry we detected that voiding improved in all patients. Both preoperative mean Qmax and mean Qavg increased, the former from 6.2 to 22.5ml/s, the latter from 4.3 to 12.4 ml/s, (p<0.001), at 12 months post-operation. Mean post-void residual volume (PVR) decreased from 89 ml before operation to 10 ml, (p<0.001), at 12 months post-operation. Mean inner urethral size increased from 3.9mm one-month post-operation to 5.4 mm, (p<0.001), 9 months’ post-operation. No recurrent US was detected in any of the patients during the time of observation (now the average time of follow-up is 86 months, ranging from 26 to 144). A follow-up with a calibration of the urethra with the last adjusted Hegar was done annually as well as on patient's request. We observed that a routine calibration with anti-scar gel preventing scar contraction after urethroplasty in the long-term is a safe and effective method of postoperative management. Patients are very motivated to continue self-calibration since they suffered a lot for a long period of time due to serious problems with voiding as well as due to the painful memory of many operations.

In the authors' opinion, BMGU as the “gold standard” in long narrowed segment and recurrent anterior US, could be enriched by calibration with anti-scar gel, as a routine postoperative procedure which may form comprehensive, broadened therapy for US. Along with BMGU - if other authors confirm the effectiveness of our method, - may become a new standard of procedure (5).

The Author
